# Optimization of a Self-microemulsifying Drug Delivery System for Oral Administration of the Lipophilic Drug, Resveratrol: Enhanced Intestinal Permeability in Rat

**DOI:** 10.34172/apb.2023.054

**Published:** 2022-07-02

**Authors:** Shahla Mirzaeei, Negar Tahmasebi, Ziba Islambulchilar

**Affiliations:** ^1^Nano Drug Delivery Research Centre, Health Technology Institute, Kermanshah University of Medical Sciences, Kermanshah, Iran.; ^2^Pharmaceutical Sciences Research Centre, Health Institute, Kermanshah University of Medical Sciences, Kermanshah, Iran.; ^3^Student Research Committee, School of Pharmacy, Kermanshah University of Medical Sciences, Kermanshah, Iran.; ^4^Department of Pharmaceutics, Faculty of Pharmacy, Tabriz University of Medical Sciences, Tabriz, Iran.

**Keywords:** Antioxidant, Intestinal permeability, Oral drug delivery, Resveratrol, Self-micro emulsifying drug delivery systems, Single-pass intestinal perfusion

## Abstract

**Purpose:**

This study aimed to formulate Resveratrol, a practically water-insoluble antioxidant in a self-microemulsifying drug delivery system (SMEDDS) to improve the solubility, release rate, and intestinal permeability of the drug.

**Methods:**

The suitable oil, surfactant, and co-surfactant were chosen according to the drug solubility study. Utilizing the design of experiment (DoE) method, the pseudo-ternary phase diagram was plotted based on the droplet size. *In vitro* dissolution study and the single-pass intestinal perfusion were performed for the investigation of *in vitro* and *in-situ* permeability for drugs formulated as SMEDDS in rat intestine using High-Performance Liquid Chromatography.

**Results:**

Castor oil, Cremophor^®^ RH60, and PEG 1500 were selected as oil, surfactant, and co-surfactant. According to the pseudo-ternary phase diagram, nine formulations developed microemulsions with sizes ranging between 145-967 nm. Formulations passed the centrifuge and freeze-thaw stability tests. The optimum formulation possessed an almost 2.5-fold higher cumulative percentage of *in vitro* released resveratrol, in comparison to resveratrol aqueous suspension within 120 minutes. The results of the *in-situ* permeability study suggested a 2.6-fold higher intestinal permeability for optimum formulation than that of the resveratrol suspension.

**Conclusion:**

SMEDDS can be considered suitable for the oral delivery of resveratrol according to the observed increased intestinal permeability, which could consequently enhance the bioavailability and therapeutic efficacy of the drug.

## Introduction

 Without doubt, oral administration of drugs is the most popular and convenient route of administration for the treatment of chronic disease. Many drugs are constantly administered orally, and it is also one of the most cost-effective systems for production besides all advantages reported for it.^1,2^ Oral absorption greatly depends on the dissolution rate in the gastric fluids.^3^ Most of the drugs have poor water-solubility and hence poor bioavailability as a major challenge for oral delivery.^4,5^ This poor bioavailability resulted from the fact that a drug cannot be absorbed from the gastrointestinal tract until it becomes soluble in gastric fluid.^6,7^

 The rate-limiting step for absorption of poorly soluble but easily permeable drugs belonging to class II of the biopharmaceutics classification system is their solubility in gastric fluids.^8^ Resveratrol is a water-insoluble, (aqueous solubility = 0.03 mg/mL) class II drug which, as an antioxidant polyphenol, exists in grapes, peanuts, and berries.^9^ This drug has been the subject of interest in recent years because of its anticancer, anti-aging, anti-diabetic, and cardio-protective effects.^10-13^ The low bioavailability of resveratrol following oral administration is due to its low solubility.^14,15^

 Novel drug delivery systems are capable of enhancing the pharmacokinetic properties and bioavailability of the drug through different strategies.^16^ Micro- and nano-emulsions are promising systems for the delivery of hydrophobic drugs.^17^ Furthermore, one of the popular novel drug delivery systems is the self-microemulsifying drug delivery system (SMEDDS) which is an isotropic mixture of oil, surfactant, and co-surfactant.^18^ When the formulation is faced with gastric fluids, it is rapidly converted to an oil-in-water emulsion by gentle movements of gastrointestinal muscles, hence called self-emulsifying.^19,20^ SMEDDS promotes drug absorption by increasing the amount of drug dissolved in the intestinal fluid. The drug loaded in SMEDDS can be absorbed by the lymphatic system bypassing the first-pass effect.^21^

 In this study, SMEEDS containing resveratrol were designed and optimized using the design of experiment (DoE) method. The most suitable materials for oral application with the lowest toxicity levels were selected as components, and formulations were subjected to further *in vitro* release and *in situ* permeability studies. Expectedly, optimized SMEDDS can elevate the intestinal permeation of resveratrol as a result of reduced droplet size and improved solubility.

## Materials and Methods

###  Materials 

 Resveratrol, castor oil, and polyethylene glycol (PEG) 1500 were purchased from Sigma-Aldrich (Germany). Cremophor^®^ RH 60 was obtained from BASF (Germany). Castor oil, methanol, ethanol, propylene glycol (PG), triethylamine, and sodium dihydrogen phosphate dodecahydrate were procured from Merck (Germany).

###  Determination of resveratrol solubility in various oils, surfactants, and co-surfactants

 The most important factors for the selection of oil, surfactant, and co-surfactant were non-toxicity for oral application, and possessing a high solubility for resveratrol^22^; hence, multiple biocompatible and non-toxic substances were subjected to the solubility study. To determine the most suitable components, according to the standard method,^23^ an excess amount of resveratrol was dissolved in 2 mL of different vehicles including castor oil, pomegranate seed oil, grape seed oil, liquid paraffin, olive oil, medium-chain triglyceride, soybean oil, glycerol, ethyl oleate, sesame oil, and sunflower oil as the candidates for oil phase, and PEG_200_, PEG_400_, PEG_600_, PEG_1500_, and PG as co-surfactants, and tween 20, tween 80, and Cremophor RH60 as surfactants. Then, provided mixtures were put in a Unimax 1010 DT shaking incubator (Heizmodul, Heidolph, Germany) at 36 ± 1°C and 100 rpm. After 24 hours, samples were centrifuged at 10 000 rpm for 10 minutes in a Hettich Zentrifugen Mikro 120. Afterward, mixtures were filtered through a 0.45 µm filter. The filtrates were then analysed by ultraviolet-visible (UV-Vis) spectrophotometer (UV-Mini 1240, Shimadzu, China) after diluting with absolute ethanol. The λ_max_ of the apparatus was set at 308 nm.

**Figure 1:**
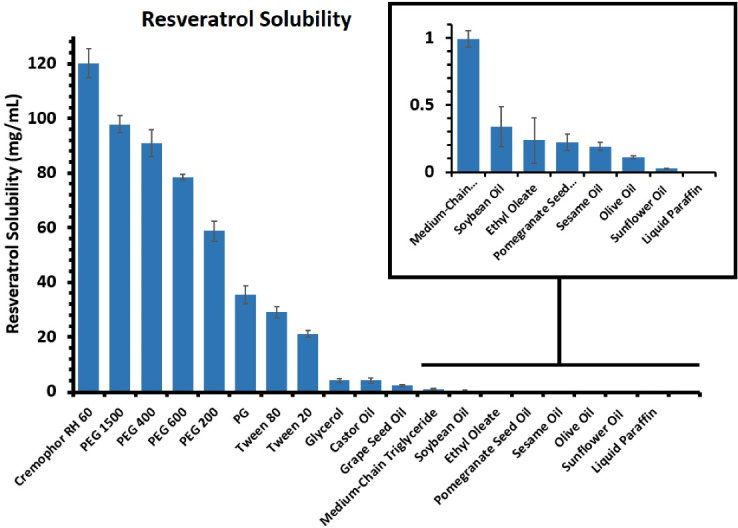
Estimated solubility of resveratrol in different oils, surfactants, and co-surfactants at 36 ± 1 °C

 The amount of resveratrol dissolved in each vehicle was calculated using the regression equation obtained by plotting a standard calibration curve for absorption versus concentration. Eventually, as shown in Figure 1, the vehicles with the maximum amount of dissolved drug were chosen for preparing the self-microemulsifying formulations. After the selection of oil, surfactant, and co-surfactant, pre-formulations were prepared and optimized based on the visual appearance and stability. The pre-formulations showing extended phase separation, turbidity, and any sign of instability were considered inappropriate and ruled out of further analysis (Table 1).

**Table 1:** The components of each pre-formulation and evaluation of microemulsion formation based on the visual appearance ( + : appropriate appearance and stability, -: inappropriate appearance and stability)

**Pre-Formulation**	**Oil (mg)**	**Co-Surfactant (mg)**	**Surfactant (mg)**	Oil:(S+Co-S)** (w:w)	**S:Co-S (w:w)**	**Appearance and stability**
P1*	100	450	450	10:90	1:1	+
P2*	100	600	300	10:90	1:2	+
P3	100	675	225	10:90	1:3	-
P4*	100	300	600	10:90	2:1	+
P5*	100	225	675	10:90	3:1	+
P6*	150	425	425	15:85	1:1	+
P7	150	566	284	15:85	1:2	-
P8	150	636	214	15:85	1:3	-
P9*	150	284	566	15:85	2:1	+
P10*	150	214	636	15:85	3:1	+
P11	200	400	400	20:80	1:1	-
P12	200	533	267	20:80	1:2	-
P13	200	600	200	20:80	1:3	-
P14*	200	267	533	20:80	2:1	+
P15*	200	200	600	20:80	3:1	+

*Optimized pre-formulations **S: Surfactant, Co-S: Co-Surfactant.

###  Construction of pseudo-ternary phase diagram

 After detection of the suitable variation range for each component in the mixture based on the result of the pre-formulation study, the DoEmethod was used for drawing a pseudo-ternary phase diagram to determine the appropriate ratios of oil, surfactant, and co-surfactant in which the microemulsions could have been developed. The advantages of the DoE method are reducing the number of required experiments and forecasting the probable interactions between variables and their impress on the final response.^24^ Utilizing Minitab^®^ software, the minimum number of required experiments (9 experiments) were designed using extreme vertices design which is a subset of DoE methods. The order of experiments was randomized to eliminate the effect of disturbing variables. The mixtures were prepared in the laboratory based on the weight ratios obtained by DoE under similar conditions and then were analysed for the globule size as the main response. The pseudo-ternary phase diagram was constructed using the obtained responses by ProSim software.

 The independent variables included the amount of oil (X_1_), surfactant (X_2_), and co-surfactant (X_3_), while globule size and polydispersity index (PDI) were chosen as the responses. The responses were analyzed based on a cubic model. The main effect of altering each factor (X_1_, X_2_, and X_3_), and the effect of simultaneous alteration of multiple factors (X_1_X_2_, X_2_X_3_, X_1_X_3_, and X_1_X_2_X_3_) were evaluated by determination of coefficients.

 Two milliliters of each formulation consisting of castor oil as oil phase, Cremophor RH60, and PEG 1500 as surfactant system were soaked in 100 mL of distilled water and gently stirred at 37°C then were undergone size analysis. The formulations with suitable globule size and PDI were chosen for further dissolution study.

###  Preparation of SMEDDS formulations

 To prepare the SMEDDS formulations, in a glass vial, 50 mg of resveratrol was dissolved in melted PEG 1500 as the co-surfactant under a magnetic stirring, while heating to 40 to 45°C, then castor oil and Cremophor RH60 were added to the solution in the appropriate weight ratios according to Table 2. Components were mixed under continuous agitation by magnetic stirring (300 rpm) for 30 min at 50°C until a homogeneous mixture was obtained.^25^ All formulations underwent similar stirring speed and agitation and were kept solidified at room temperature after preparation until further analysis.

**Table 2:** The components, size, and polydispersity index of optimized formulations

**Formulation**	Drug* (mg)	**Oil (%w/w)**	**CO-Surfactant (%w/w)**	**Surfactant (%w/w)**	**Size (nm)**	**PDI**
F1	50	10	80	10	357 ± 50	0.540 ± 0.116
F2	50	20	70	10	967 ± 92	0.711 ± 0.134
F3	50	22	63	15	693 ± 32	0.648 ± 0.125
F4	50	18.125	78.625	3.25	710 ± 70	0.697 ± 0.061
F5	50	18.625	73.625	7.75	216 ± 12	0.396 ± 0.105
F6**	50	10	70	20	145 ± 9	0.116 ± 0.059
F7	50	10	65	25	759 ± 63	0.789 ± 0.124
F8	50	25	60	15	898 ± 45	0.712 ± 0.140
F9	50	20	60	20	217 ± 17	0.410 ± 0.111

*Drug amount (mg) per 500 mg of formulation. **Optimized formulation selected for *in situ *study.

###  Physical stability 

 Optimized formulations were analysed for stability using the standard centrifuge and freeze-thaw test.^26^ Through the centrifuge test, formulations were centrifuged (Rotofix 32, Hettich Zentrifugen, Germany) for 30 minutes at 10 000 rpm, then were visually observed for alteration of visual appearance like phase separation, sedimentation, creaming, and cracking. If the formulations passed this test, they were subjected to freeze-thaw cycles.

 To perform the freeze-thaw cycles, each optimized SMEDDS formulation was stored in a glass vial at -4°C for 24 hours followed by keeping at 40°C for another 24 hours. The cycle was repeated three times. Finally, samples were visually inspected for instability, and the size changes were evaluated by a zeta-sizer.

###  Compatibility with capsules

 To determine the compatibility of resveratrol-loaded SMEDDS with capsules, formulations were kept for three months at room temperature in hard gelatin capsules to evaluate any leakage and changes.

###  Droplet size analysis

 One gram of SMEDDS formulations was dispersed in 250 mL of distilled water under magnetic stirring (about 100 rpm) at 37°C to simulate the GI temperature and agitation rate. Photon correlation spectroscopy was performed using a zeta-sizer for analyzing of droplet size and PDI of each dispersion.

###  Fourier-transform infrared spectroscopy (FTIR)

 To investigate any incompatibility between the drug and other components, an FTIR study was performed.^27^ One milligram of solidified SMEDDS formulation, each component, and resveratrol were separately compressed with potassium bromide into a disk by the manual press under 10 tons pressure. The FTIR spectra of samples were recorded in a 4000-400 cm^-1^ scanning range using an IR prestige 21 FTIR spectrophotometer (Shimadzu, Japan).

###  In vitro drug dissolution study

 USP XXII apparatus type I was used for determining *in vitro* drug release.^28^ The rate and temperature of the apparatus were set at 50 rpm and 37°C to simulate GI conditions. The vessels were filled with 900 mL of HCl (pH = 1.2) as the dissolution medium. Each formulation was put in a hard gelatin capsule and then placed in the baskets and the device was run. Sampling was done in determined time intervals. In each sampling, 5 mL of dispersed media were taken and an equal volume of fresh medium was replaced immediately to preserve sink conditions. Sampling was done 15, 30, 60, and 120 minutes after beginning. Afterward, samples were filtered through a 0.45 µm filter and assayed with a UV–Vis spectrophotometer at λ_max_ of 308 nm.

###  Determination of in situ intestinal permeability with single-pass intestinal perfusion (SPIP) method 

 There are different models for *in situ* intestinal permeation studies. One of the most important models is regional *in situ* perfusion or SPIP.^29^ In this method male Wistar rats of about 200-300 g weight were chosen and kept in the laboratory for 18 hours fasting with free access to water. Each animal was anesthetized with an intraperitoneal injection of ketamine. To provide the appropriate temperature, the animal was placed on an electric pad during the examination. A rat intestinal segment of about 10 cm was cannulated on both ends. To wash the intestinal contents, a normal saline solution was passed through the cannulated segment for 10 minutes. The perfusion solutions (dispersion of drug-loaded SMEDDS formulation or aqueous drug suspension in phosphate buffer) were then passed through the cannula at a rate of 0.2 mL/min. No samples were withdrawn in the first 30 minutes to reach a steady-state, then samples (2 mL) were collected every 10 minutes for about 90 minutes. At the end of the experiment, the length of the cannulated part was measured and then the animal was sacrificed according to ethical guidelines.^30,31^ Samples were stored at -20°C until analysed with high-performance liquid chromatography (HPLC). The whole process was approved by the ethical committee.

 The chromatography was performed on a 5 μm, PerfectSil ODS-2, (250 mm × 4 mm), analytical column (MZ-Analysentechnik, Germany). The mobile phase consisted of a 90:10 v/v ratio of methanol and triethylamine 0.1%w/v aqueous solution mixture (pH adjusted to 3.0 with phosphoric acid) with a flow rate of 1 mL/min. The injection took place manually using a Rheodyne 7725i injector, for a 20 μL sample loop. The detector was set at 308 nm and the retention time was 2.8 minutes. To determine the concentration of resveratrol in the samples, a calibration curve was drawn. Finally, the calibration curve was plotted, and the regression equation was used to assay the samples. The effective permeability was calculated using the following formula:


(Equation 1) Peff=−Qin LnCoutCin2πlr

*Peff=effective permeability*

*C_in_=drug concentration in the injected solution*

*C_out_=drug concentration in the sample solution*

*Qin=the flow rate of drug injection in the intestine of rats*

*2πlr=intersection area of drug solution with intestine*

###  Photostability Study

 To examine the photostability of the drug, SMEDDS formulation and free drug solution were exposed to UVA radiation at 365 nm for 16 min at a distance of 65 mm according to a method established by Coimbra et al in a previous study^32^; then, the drug content of formulations was examined by HPLC. The percentage of resveratrol that remained intact in formulations was compared to evaluate the photostability of the drug that was loaded in the formulation.

## Results and Discussion

###  Solubility study

 As mentioned earlier, resveratrol is a water-insoluble drug with a solubility of 0.03 mg/mL in water.^18^ As a result, the selection of components with high solubility can greatly reduce the likelihood of its deposition in the microemulsion system. In addition, this issue is also especially important in terms of the effect on drug delivery. Finally, castor oil was the best solvent for the drug. Cremophor RH60 and PEG 1500 were respectively selected as surfactant and co-surfactant because of the ability to solve the largest amount of drug and acquiring greater stability compared to other compounds. The results of the solubility study are presented in Figure 1.

###  Construction of pseudo-ternary phase diagram

 The pre-formulations were prepared using castor oil, Cremophor RH60, and PEG 1500 as oil, surfactant, and co-surfactant on a magnetic stirrer at 35 to 40°C and 100 rpm for 15 minutes. Based on the visual appearance and stability of pre-formulations, P1, P2, P4, P5, P6, P9, P10, P14, and P15 were selected as the optimized pre-formulations (Table 2). These pre-formulations give information about the suitable range of components as the variables. The suitable range for the variation of oil content was between 10-25% w/w of the formulation while it was supposed to be 60-80% for co-surfactant and 3-25% for surfactant.

**Figure 2:**
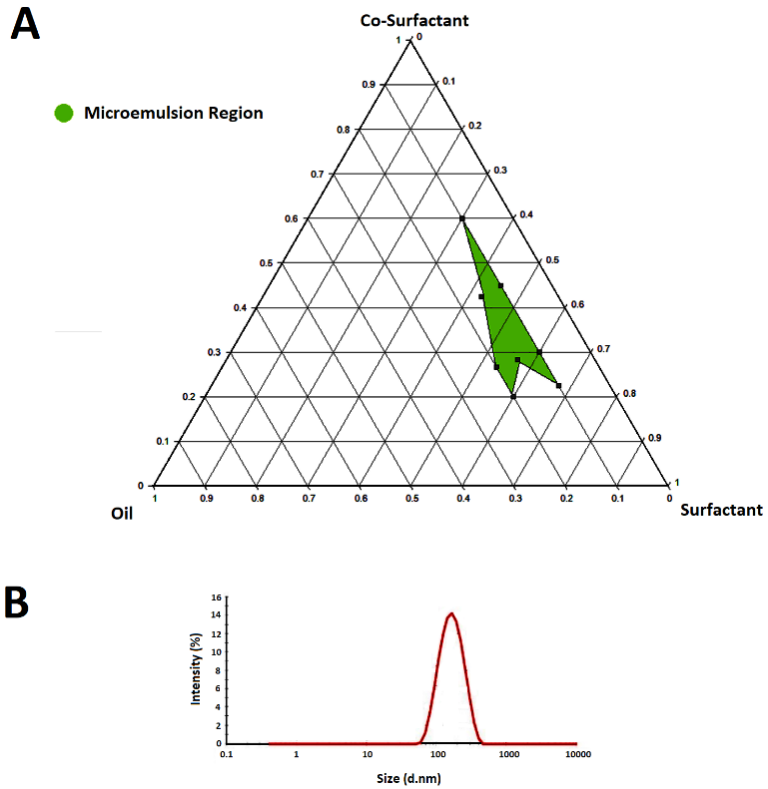
The pseudo-ternary phase diagram (A) constructed by ProSim software for a mixture of castor oil, Cremophor RH60, and PEG1500 respectively as oil, surfactant, and co-surfactant. Size distribution (B) of formulations investigated by a zeta-sizer at 25°C

 After obtaining the required ratios using the DoE method, the formulations were prepared and analysed for globule size and PDI, and the pseudo-ternary phase diagram was plotted by ProSim software based on the size response (Figure 2A). The analysis of response Minitab^®^ software indicated suitable randomization. The surfactant to the co-surfactant ratio (Smix) was kept lower than 0.5 (w/w ratio) in all formulations to avoid the depletion of the microemulsion region. Increasing this ratio can lead to a higher viscosity that resists the formation of globules with lower size because of higher surfactant concentration. Increasing co-surfactant concentration enhances the fluidity of oil to the hydrophobic region of the surfactant molecule aiding the formation of microemulsions. All formulations (F1-F9) showed a size in the micron range while F6 possessed the best particle size and PDI (Figure 2B).

 As the *P* value was measured to be more than 0.05 for both responses in each model, the values were not completely fitted in any model but the cubic model showed higher regression. Regression coefficient R^2^ values for size (Y_1_) and PDI (Y_2_) responses were calculated to be 0.9045 and 0.8431. Table 3 represents the coefficients of each factor and the interactions. It is obvious that there is a higher correlation between variables and size response than PDI. The observed size values of various emulsions varied from 145 to 967 nm. Equation 2 and 3 indicates the effect of variables on size and PDI values. Each independent variable shows the main effect on size response; the negative coefficient value of X_1_X_2_, X_1_X_3,_ and X_2_X_3_ indicates the antagonist effect of simultaneous alteration of these factors on size while the positive coefficient of X_1_, X_2_, and X_3_ showed an agonist effect of them on the size response. The surfactant alteration shows an antagonist effect on PDI while oil and co-surfactant display an agonist effect on this response.

**Table 3:** The coefficients obtained for the effect of various factors on the responses

**Variables **	**Coefficients **	
	**Size (Y**_1_**)**	**PDI (Y**_2_**)**
X_1*_	16946.0	15.5542
X_2*_	807.6	-1.2976
X_3*_	1306.5	1.2010
X_1_X_2_	-1204.1	-1.0798
X_1_X_3_	-283.8	-0.2609
X_2_X_3_	-116.3	-0.0840
X_1_X_2_X_3_	19.8	0.0182

*X_1 _(Oil), X_2_ (Surfactant), X_3_ (Co-surfactant)


(Equation 2) $$Y_{1}=16946.0X_{1}+807.6X_{2}+1306.5X_{3}-1204.1X_{1}X_{2}-283.8X_{1}X_{3}-116.3X_{2}X_{3}+19.8X_{1}X_{2}X_{3}$$

(Equation 3) $$Y_{2}=15.5542X_{1}-1.2976X_{2}+1.2010X_{3}-1.0798X_{1}X_{2}-0.2609X_{1}X_{3}-0.0840X_{2}X_{3}+0.0182X_{1}X_{2}X_{3}$$

###  Physical stability

**Figure 3:**
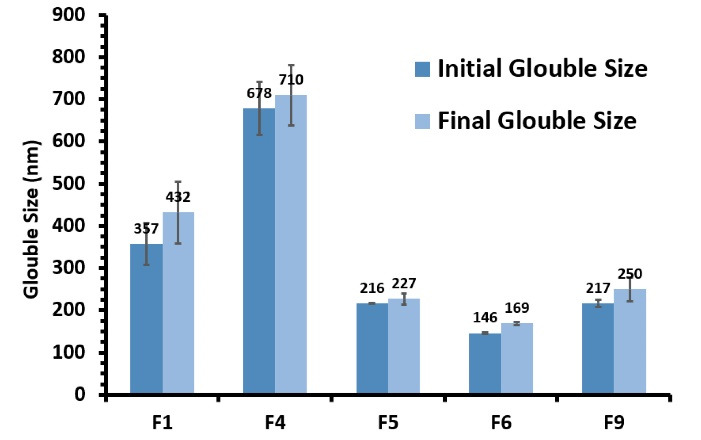
The Globule size changes of F1, F4, F5, F6, and F9 formulations after three replicates of freeze-thaw cycles with 48 h incubation at each temperature (-20 to + 25 °C) during the stability test

 SMEDDSs should form stable microemulsions after interaction with an aqueous medium. This can be achieved by the optimized ratio of components. Among all the formulations, F1, F4, F5, F6, and F9 did not show any phase separation during the centrifuge test and entered the freeze-thaw cycles. None of these optimized formulations (F1, F4, F5, F6, and F9) demonstrated any significant sign of instability including creaming, phase separation, and rise in particle size showing appropriate stability.^33^ Figure 3 demonstrates the size changes from the baseline of prepared formulation during the freeze-thaw test. Importantly, there was no significant increase in the size of the microemulsion. As a result, these five formulations, which had good thermodynamic and physical stability, were subjected to dissolution study.

###  Compatibility with capsule

 Optimized SMEDDS formulations were weighed and loaded inside hard gelatin capsules for three months at room temperature and evaluated for the leakage of contents. None of the formulations showed any leakage out of the capsule, and all of the formulations were subjected to the stability test.

###  Droplet size analysis 

 Analysing the size and PDI of the F6 as the optimized SMEDDS formulation consisting of 10% w/w oil, 20% w/w surfactant, and 70% w/w co-surfactant showed the mean particle size of 145 ± 9 nm and the PDI of 0.116 ± 0.059 both considered appropriate (Figure 2B). The prepared emulsion as a result of the dispersion of F6 in the aqueous medium could be considered a nanoemulsion due to the mean size below 200 nm.

 The nanoemulsion was developed utilizing the spontaneous emulsification technique. In this technique, the size is reduced by the selection of the appropriate oil, surfactant, and cosurfactant at the optimum concentration as there is no control on the emulsification phase in SMEDDS formulations and based on the fact that the emulsification occurs inside the GI system with a predetermined environment.^34^ This method is classified as a low-energy emulsification method.^35^

###  FTIR

**Figure 4:**
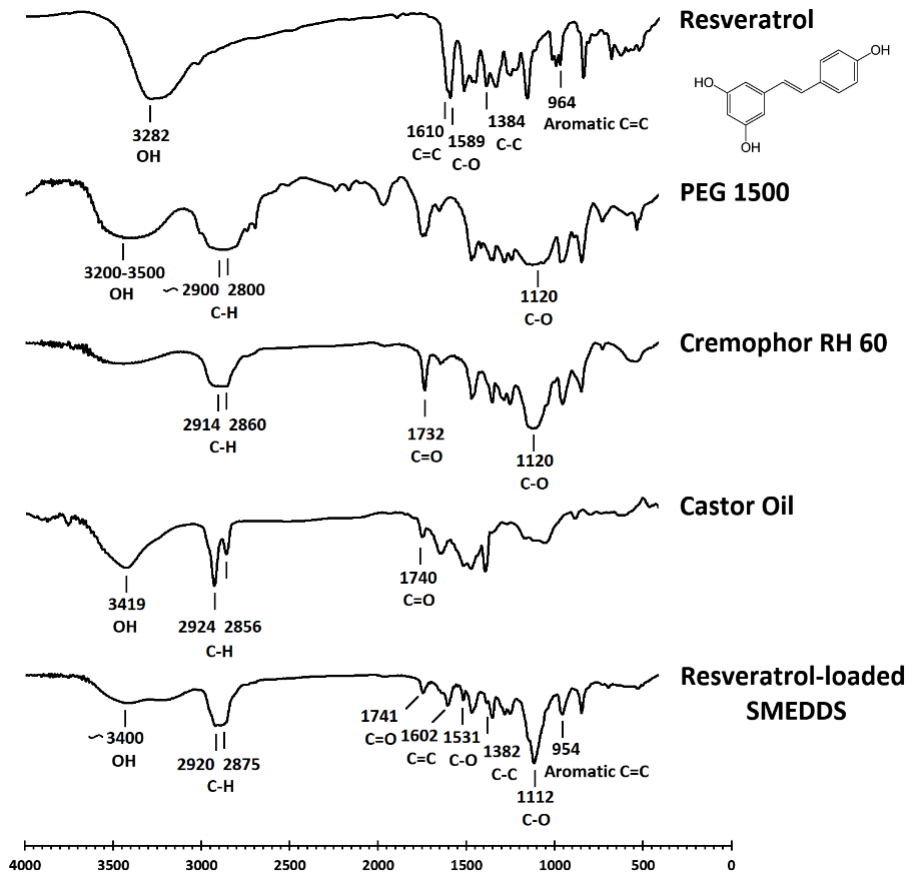
FTIR Spectra of resveratrol, PEG_1500_, Cremophor^®^ RH60, castor oil, and solidified SMEDDS

Figure 4 displays the results of the FTIR study. Pure resveratrol showed five characteristic peaks at 3282, 1610, 1589, 1384, and 964 cm^-1^ attributed to phenolic OH bond stretching, C = C olefinic band, C-O stretching vibration, C-C stretching, and aromatic C = C bond stretching, respectively.^36^ According to the SMEDDS spectrum, the peaks at 2920 and 2875 cm^-1^ are attributed to the asymmetrical and symmetrical stretching of C-H bonds in Cremophor RH60, PEG_1500_, and castor oil.^37,38^ At 1112 cm^-1^, there is a sharp peak which is assigned to the C-O stretching of PEG_1500_ and Cremophor RH60.^39^ The characteristic peaks of castor oil appear around 3400 and 1741 cm^-1^ corresponding to OH and C = O stretching, respectively.^37^ Most of the characteristic peaks concerning the functional groups of resveratrol were widened in the SMEDDS spectrum presumably due to the loss of crystallinity of the drug that was loaded in the carrier.^40^ Also, the decreased intensity of some peaks is attributed to the hydrogen bonds formed by virtue of resveratrol dissolving in formulation excipients.^41^ According to the obtained results, characteristic peaks of resveratrol appear in the SMEDDS spectrum. Accordingly, no major changes were observed for resveratrol peaks, which indicates drug-excipients compatibility.

###  In vitro drug dissolution study

 Capsules loaded with self-microemulsifying formulations released resveratrol faster than the ones filled with an equal amount of plain drug by releasing more than 70% of their drug content during the first 30 minutes. All capsules were disintegrated in less than 1 min but the dissolution occurred in a longer duration. The occurrence of the emulsification phenomenon following the disintegration of SMEDDS-loaded capsules led to the faster dissolution of the drug in the dissolution medium. Many previous studies proved that formulating a drug as SMEDDS would improve the solubility rate.^42-44^ After 1 hour, capsules filled with F1, F4, F5, F6, F9, and plain drug released 92.39 ± 0.66%, 94.27 ± 4.88%, 84.85 ± 7.11%, 94.05 ± 2.41%, 79.82 ± 0.12%, and 36.38 ± 0.18% of their drug content.

**Figure 5:**
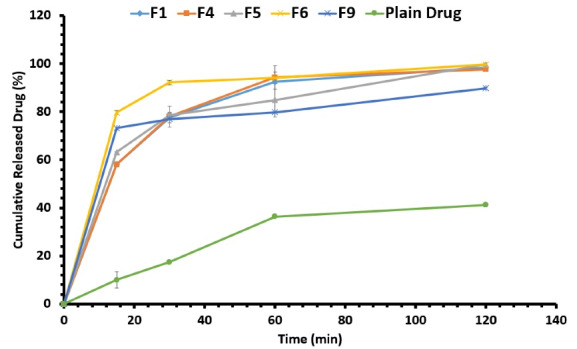
*In vitro *release profiles of different SMEDDS formulations along with the plain drug estimated by the dissolution test in USP type II apparatus filled with HCl 0.1 N at 37°C

 F6 showed the fastest release profile by releasing an almost 8-folds higher amount of drug in the first 15 minutes compared to the plain drug. As a result, the F6 formulation was selected as the optimal formulation. In Figure 5, the cumulative percentage of released resveratrol from the formulations can be seen. The faster and enhanced *in vitro* release of drug from formulations could be due to the smaller globule size leading to a higher surface-to-volume ratio and thus higher diffusion in the release medium. F6 formulation with the smallest globule size demonstrated the fastest release rate confirming this theory. Similar studies suggested the same reason for enhanced *in vitro* dissolution and higher cumulative release of drug from SMEDDS formulations; for example, Xu et al claimed that enhanced *in vitro* dissolution of [6]-Gingerol from SMEDDS formulation compared to free drug, was probably due to its small droplet size.^45^ In another study, the smaller particle size of microemulsions that were formed following the exposure of SMEDDS formulation to the aqueous medium, was presumed to be the main reason behind the higher cumulative release of 6-shogaol.^46^

 In a similar study, a self-emulsifying system was prepared to enhance the dissolution rate of fenofibrate, which showed an improved dissolution compared to the plain drug.^47^ Bandi et al reported an enhanced dissolution rate of risperidone by the incorporation of this drug into a self-emulsifying system compared to the marketed tablets.^48^ A self-nanoemulsifying system was prepared using Capryol^®^ 90, Cremophore^®^ EL, and Transcutol^®^ HP for the delivery of docetaxel reported to increase drug dissolution compared to the drug powder.^49^

###  In situ intestinal permeability: SPIP method 

**Figure 6:**
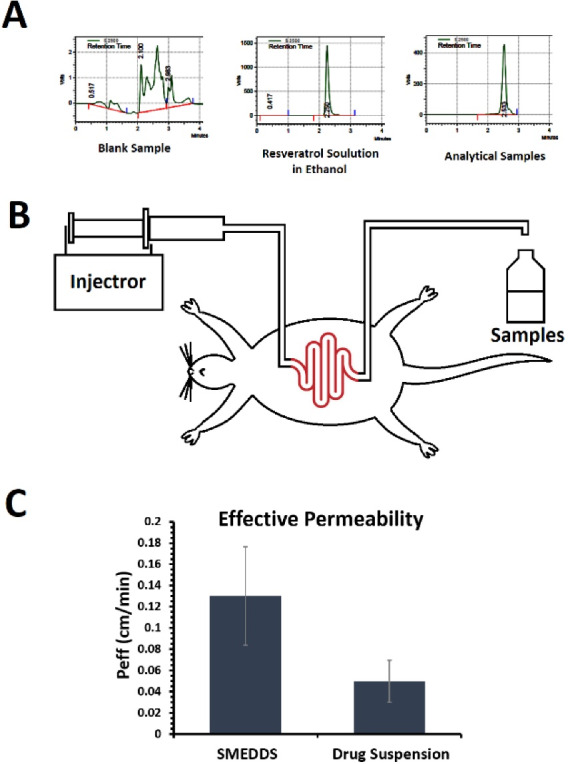
The HPLC peaks (A) obtained for different samples. Schematic presentation of SPIP study process (B) for evaluation of *in situ *intestinal permeability. *In situ* effective intestinal permeability (C) estimated for SMEDDS formulation (F6) and resveratrol aqueous suspension using the SPIP method

 The resveratrol calibration curve was plotted using the area under the peak curve obtained from different resveratrol concentrations with HPLC. The HPLC peak of the blank, resveratrol dissolved in ethanol, and one of the samples in the SPIP method were shown in Figure 6A. The mean effective intestinal permeability was obtained for resveratrol in rat intestines for the experimental and control groups based on the SPIP method (Figure 6B). It showed that the intestinal permeability of resveratrol increased from 0.0498 cm/min in the control group receiving the drug aqueous suspension to 0.1302 cm/min in the experimental group receiving F6 formulation suspended in water as shown in Figure 6C.

 This result is in accordance with a previous study on intestinal absorption of resveratrol.^50^ In the mentioned study which designed self-nanoemulsifying drug delivery system (SNEDDS) for the delivery of resveratrol, enhanced bioavailability and permeability parameters were observed for the SNEDDS compared to the pure drug. The incorporation of resveratrol into SMEDDS formulations has resulted in a significant increase in its intestinal permeability which may be due to the enhancement in the solubility of resveratrol in the GI tract. Under the mild agitations provided by the gastrointestinal motility, SMEDDS formulations can form microemulsions with fine droplet sizes after dilution with aqueous GI fluids.^51^ The small droplet size of the dispersed SMEDDS formulations may lead to improved drug absorption because wider surface area SMEDDS can easily form chylomicrons and consequently can be absorbed via the lymphatic system along with facilitating trans and paracellular drug transport.^52^ Furthermore, non-ionic surfactants like Cremophor RH60 are known to act as absorption enhancers.^53^

 Each of the above-mentioned mechanisms could be considered as a potential reason to explain the role of SMEDDS formulations in the observed enhancement in the intestinal permeability of resveratrol as a lipophilic molecule. Besides, its ability to distinguish such a significant enhancement in the intestinal permeability of SMEDDS-loaded drugs compared to the free drug makes the SPIP a reliable method to assess the intestinal effective permeability of SMEDDS formulations.

 A similar study reported a 2-4 folds higher amount of permeated drug through the isolated intestinal mucosa of rats by a self-nanoemulsifying powder of risperidone compared to the marketed formulation and pure drug powder.^48^ A self-emulsifying system based on castor oil showed improved pharmacokinetics for the antipsychotic drug Levosulpiride.^54^ Loading the lercanidipine hydrochloride in SMEDDS comprised of Capmul MCM C8, brij35, cremophor EL, and propylene glycol created improved dissolution and permeation of the drug.^26^ Furthermore, another study reported an improved bioavailability and dissolution for CoQ10 loaded in a SNEDDS system.^23^

 Other methods have also been used to evaluate drug-loaded SMEDDS. Jaisamut et al developed a liquid SMEDDS to improve the oral bioavailability of quercetin and resveratrol. To evaluate the efficacy of the developed formulation, the *in vivo* pharmacokinetics of the drugs were investigated following the oral administration of the formulation in rats. The 3- to 9-folds higher area under the curve of quercetin and resveratrol compared to the pure drugs demonstrated the efficacy of SMEDDS to enhance the oral bioavailability of these drugs.^55^ Another study developed a phospholipid complex and SMEDDS of resveratrol. In this study, the efficacy of the drug delivery system was evaluated by investigating pharmacokinetics in rats, which indicated an enhanced oral bioavailability.^56^

###  Photostability study

 Resveratrol is an antioxidant with low photostability; hence the major reason for resveratrol instability is photodegradation.^57^ One of the main objectives for formulating resveratrol in novel carriers including liposomes, nanoemulsions, SMEDDS, etc is enhancing its photostability. Multiple studies pointed to the ability of such systems in enhancing the stability of drugs like resveratrol.^57,58^ In a similar study, loading of resveratrol in liposome carrier protected 70% of the drug from degradation after 16 min of UV exposure while 90% of the free drug was degraded in the same conditions.^32^ The results of the photostability study indicated that drug content in the SMEDDS formulation was 85.35 ± 1.41% after 15 minutes exposure to UV light while this value for the free drug solution was 15.76 ± 0.88%. These results confirmed an enhanced photostability of the drug after loading in SMEDDS formulation. Of note, all formulations were filled in dark-colored capsules which protect them from light and air exposure, and kept in amber glass containers to avoid photodegradation of the drug. Moreover, the prepared SMEDDS formulation was in solidified form in the present study which could reduce the UV exposure compared to microemulsion formulation according to previous studies.^59^ Owing to the photoprotective nature of developed SMEDDS formulations along with the solidified form with lower light permeability, long-term stability is predicted for formulations.

## Conclusion

 SMEDDSs, by definition, are systems with thermodynamic stability that spontaneously formed microemulsions by exposing them to a physicochemical aqueous medium and are suitable for enhancing the delivery of poorly water-soluble drugs. In this study, SMEDDS for resveratrol, an antioxidant hydrophobic polyphenol with a wide range of benefits for human health, was prepared. After the selection of suitable vehicles to find the microemulsion region, the pseudo-ternary phase diagram was plotted based on globule size after dispersion in PBS. To evaluate the stability of formulations, centrifuge tests, and freeze-thaw cycles were carried out, showing the stability of F1, F4, F5, F6, and F9 formulations. *In vitro* release profiles of F1, F4, F5, F6, F9, and the plain drug loaded in hard gelatin capsules were observed. In this connection, F6 indicated the fastest release by 8-fold compared to a plain drug, hence selected for *in situ* study. *In situ* study was carried out by SPIP technique. The intestinal permeability of resveratrol for the F6 was 3 times higher compared to that of drug suspension. It can be concluded that SMEDDS is a suitable carrier for enhancing the solubility, intestinal permeability, and thus oral delivery of resveratrol.

## Acknowledgments

 The authors would like to thank the Research Council of Kermanshah University of Medical Sciences (Grant Number: 990456) for the financial support of this work. Also, faithfully thank Rahesh Daru Novin knowledge-based company for providing materials and equipment.

## Competing Interests

 The authors declared no conflict of interest in this study.

## Ethical Approval

 The whole procedure was approved by the Ethics Committee (approval number: IR.KUMS.REC.1397.1051), Kermanshah University of Medical Sciences, Kermanshah, Iran.
